# Effect of berberine on global modulation of lncRNAs and mRNAs expression profiles in patients with stable coronary heart disease

**DOI:** 10.1186/s12864-022-08641-2

**Published:** 2022-05-26

**Authors:** Ye-Chen Han, Hong-Zhi Xie, Bo Lu, Ruo-Lan Xiang, Jing-Yi Li, Hao Qian, Shu-Yang Zhang

**Affiliations:** 1 Department of Cardiology, Peking Union Medical College Hospital, Chinese Academy of Medical Sciences and Peking Union Medical College, No.1 Shuaifuyuan, Dongcheng District, Beijing, 100730 China; 2 Department of Gastroenterology, Peking Union Medical College Hospital, Chinese Academy of Medical Sciences and Peking Union Medical College, Beijing, 100730 China; 3 Department of Physiology and Pathophysiology, Peking University School of Basic Medical Sciences, Beijing, 100191 China

**Keywords:** Berberine, Stable coronary heart disease, lncRNA, mRNA

## Abstract

**Background:**

Berberine (BBR) is an isoquinoline alkaloid found in the Berberis species. It was found to have protected effects in cardiovascular diseases. Here, we investigated the effect the regulatory function of long noncoding RNAs (lncRNAs) during the treatment of stable coronary heart disease (CHD) using BBR. We performed microarray analyses to identify differentially expressed (DE) lncRNAs and mRNAs between whole blood samples from 5 patients with stable CHD taking BBR and 5 no BBR volunteers. DE lncRNAs and mRNAs were validated by quantitative real-time PCR.

**Results:**

A total of 1703 DE lncRNAs and 912 DE mRNAs were identified. Kyoto Encyclopedia of Genes and Genomes pathway analysis indicated DE mRNAs might be associated with mammalian target of rapamycin and mitogen-activated protein kinase pathway. These pathways may be involved in the healing process after CHD. To study the relationship between mRNAs encoding transcription factors (DNA damage inducible transcript 3, sal-like protein 4 and estrogen receptor alpha gene) and CHD related de mRNAs, we performed protein and protein interaction analysis on their corresponding proteins. AKT and apoptosis pathway were significant enriched in protein and protein interaction network. BBR may affect downstream apoptosis pathways through DNA damage inducible transcript 3, sal-like protein 4 and estrogen receptor alpha gene. Growth arrest-specific transcript 5 might regulate CHD-related mRNAs through competing endogenous RNA mechanism and may be the downstream target gene regulated by BBR. Verified by the quantitative real-time PCR, we identified 8 DE lncRNAs that may relate to CHD. We performed coding and non-coding co-expression and competing endogenous RNA mechanism analysis of these 8 DE lncRNAs and CHD-related DE mRNA, and predicted their subcellular localization and N^6^-methyladenosine modification sites.

**Conclusion:**

Our research found that BBR may affect mammalian target of rapamycin, mitogen-activated protein kinase, apoptosis pathway and growth arrest-specific transcript 5 in the process of CHD. These pathways may be involved in the healing process after CHD. Our research might provide novel insights for functional research of BBR.

**Supplementary Information:**

The online version contains supplementary material available at 10.1186/s12864-022-08641-2.

## Background

Cardiovascular disease is a serious threat to human health. Coronary heart disease (CHD) is a leading cause of morbidity and mortality worldwide and also a major socioeconomic burden [1]. CHD has become a serious disease affecting human life quality and span. Due to the great progress of modern science, technology and medicine in recent decades, the prognosis of CHD has been improved. However, the decline of cardiac function after CHD will still lead to heart failure and poor prognosis [2].

Berberine (BBR) is an isoquinoline alkaloid isolated from berberis species, and is an essential component of the Chinese herbal medicine called coptis. BBR is a nonprescription drug for diarrhea, and also widely used for treatment of cardiovascular diseases [3]. BBR attenuates the development of atherosclerosis, suppresses ischemic arrhythmias, improves cardiovascular hemodynamics, and reduces hypertension [4]. Studies have shown that BBR may prevent reperfusion injury / myocardial ischemia through anti-inflammatory and antioxidant effects. It can increase superoxide dismutase level, decrease apoptosis, diminish lactate dehydrogenase and serum creatine kinase levels, which are associated with activation of SIRT1 signaling [4]. Combination of BBR and trimetazidine can increase the content of nitric oxide in blood and promote endothelium-dependent dilation function of brachial arteries, which is helpful in endothelial function of patients with CHD combined with primary hypertension [5]. In the rat model of cardiac hypertrophy by constriction of the abdominal aorta, BBR has been reported to attenuate cardiomyocyte enlargement and pressure-overloaded myocardial hypertrophy through enhancing autophagy and down-regulating the expression of lncRNA myocardial infarction associated transcript (MIAT) [6].

Long noncoding RNAs (lncRNAs) are a class of noncoding RNAs longer than 200 nucleotides [7]. LncRNAs are involved in a wide range of biological processes, such as transcriptional and post transcriptional regulation. LncRNAs also have been found to be widely involved in the process of CHD. LncRNA FAF was lowly expressed in the serum of patients with CHD. It might be an independent risk factor for poor prognosis of patients with CHD [8]. LncRNA CAIF inhibits autophagy and alleviates myocardial infarction (MI) by blocking p53-mediated myocardin transcription [9]. Sun et al. found that lnc-MICALL2-2 is up-regulated in CHD subjects. Knockdown lnc-MICALL2-2 subsequently confers protection against early pathological processes of oxidized low-density lipoprotein-induced CHD [10]. The level of lncRNA H19 is significantly decreased in the heart tissue of Sprague-Dawley rats undergoing MI surgery. LncRNA H19 improves MI-induced myocardial injury and cardiac remodeling by regulating miR-22-3p/KDM3A pathway [11]. BBR has been found to regulate the expression of lncRNAs in a variety of tissues. Cao et al. found that BBR alleviated inflammation in ischemic stroke through the Malat1/miR-181c-5p/HMGB1 axis in mice pretreated with BBR before the 60-min MCAO surgery [11]. BBR enhanced autophagy by inhibiting the expression of lncRNA MIAT, and significantly inhibited myocardial hypertrophy and cardiomyocyte enlargement caused by constriction of abdominal aorta [6].

At present, the protective effect of BBR following stable CHD is still lacking research. Its clinical application is not widespread, and its molecular mechanism is still unknown. Here, we want to explore the role of lncRNA in protective effect of BBR in cardiovascular diseases. In this study, microarray analysis was used to observe the changes of lncRNAs and mRNAs expression profiles in patients with stable CHD taking BBR. Our research found that BBR may affect mammalian target of rapamycin (mTOR), mitogen-activated protein kinase (MAPK), apoptosis pathway and growth arrest-specific transcript 5 (GAS5) in the process of CHD. These pathways may be involved in the healing process after CHD. It is an attempt to further analyze the molecular mechanism of BBR and provide new ideas for the clinical treatment of stable CHD.

## Results

### Differential expression of lncRNAs and mRNAs

**Fig. 1:**
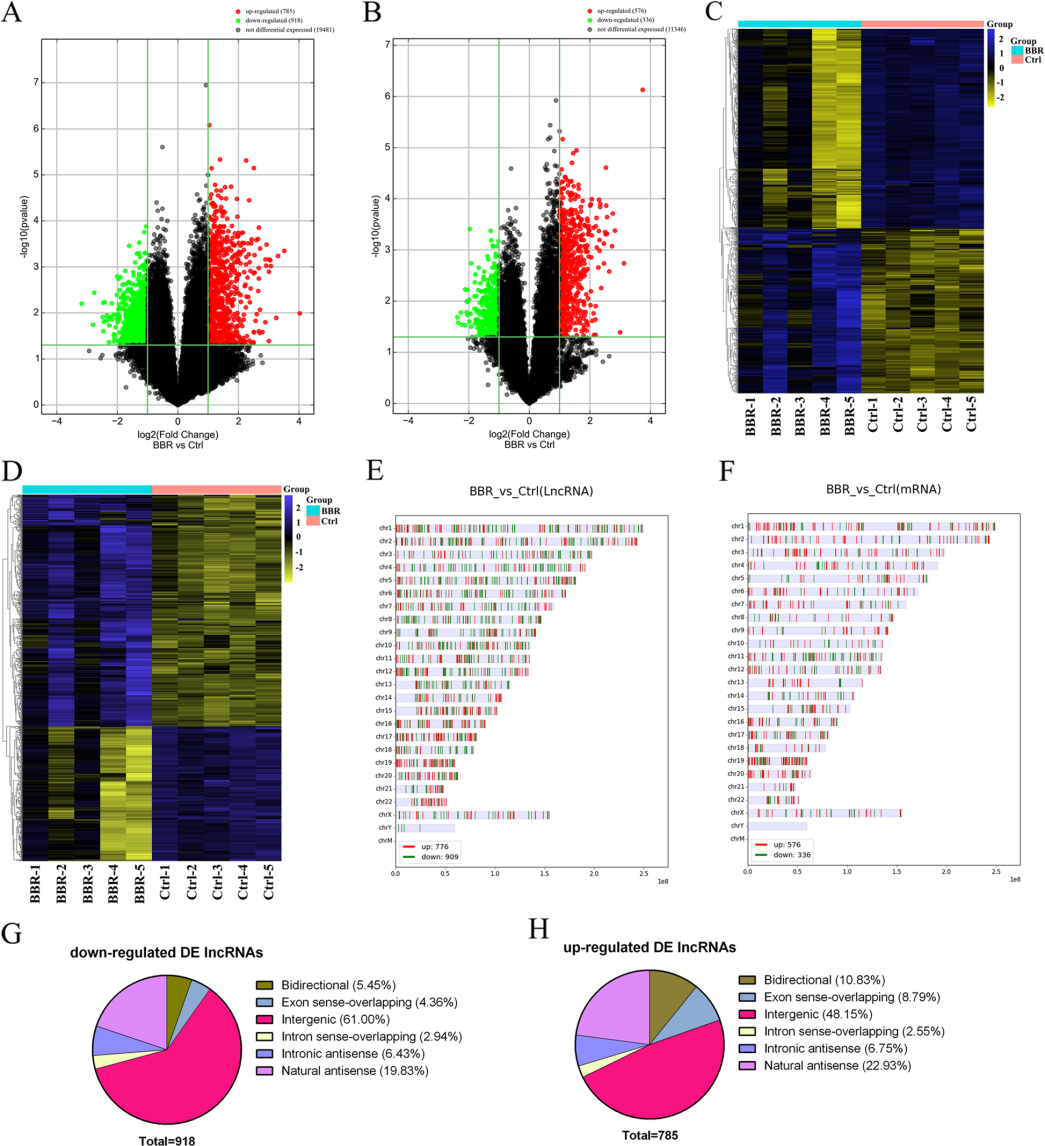
Identification of DE lncRNAs and mRNAs. Volcano plots presenting differences in the expression of lncRNAs (**A**) and mRNAs (**B**) between the BBR and control groups. Heatmaps showing the expression profiles of the top DE lncRNAs (**C**) and mRNAs (**D**). Chromosome distribution of DE lncRNAs (**E**) and mRNAs (**F**). Up-regulated (**G**) and down-regulated (H) DE lncRNAs counts and categorizations distribution. DE, differentially expressed; BBR, berberine; Ctrl, control

Human lncRNA plus mRNA microarray v5.0 was used to detect the lncRNAs and mRNAs expression profile in whole blood samples. A total of 1703 differentially expressed (DE) lncRNAs (785 up-regulated and 918 down-regulated lncRNAs) and 912 DE mRNAs (576 up-regulated and 336 down-regulated mRNAs) were detected based on fold change > 2, *P* < 0.05. Volcano plots were used to visualize the DE lncRNAs and DE mRNAs (Fig. 1A and B). Hierarchical clustering heatmaps based on their fold-changes are shown in Fig. 1C and D. The distribution of DE lncRNAs and mRNAs in chromosomes is shown in Fig. 1E-F. Figure 1G-H showed the proportion of various type of DE lncRNAs.

The data have been deposited in NCBI’s Gene Expression Omnibus and are accessible through GEO Series accession number GSE186019.

### Analysis of functional role of enriched DE mRNAs genes

**Fig. 2:**
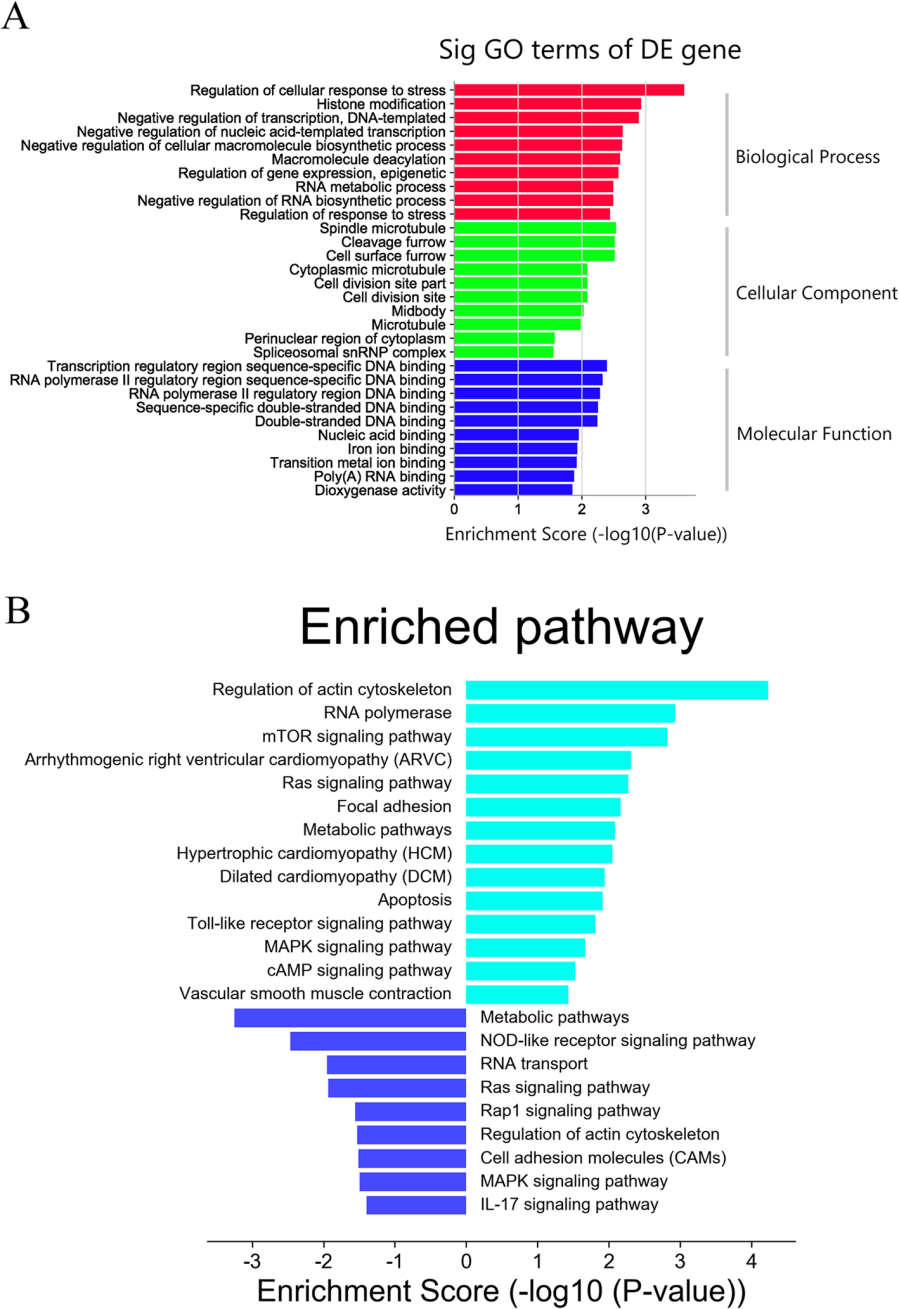
GO and KEGG pathway analyses of the DE mRNAs. **A** Top 10 terms from the GO analysis of DE mRNAs. **B** KEGG pathway analysis of up-regulated and down-regulated DE mRNAs. Left and right terms represented up-regulated and down-regulated DE mRNAs enriched pathways associated with CHD. GO, Gene Ontology; KEGG, Kyoto Encyclopedia of Genes and Genomes; DE, differentially expressed

We performed Gene ontology (GO) analysis to determine the functional significance of DE mRNAs. The most highly enriched GO terms were “Regulation of cellular response to stress (BP),” “Spindle microtubule (CC),” and “Transcription regulatory region sequence-specific DNA binding (MF)” (Fig. 2A).

The KOBAS v3.0 database was used to perform Kyoto Encyclopedia of Genes and Genomes (KEGG) [12–14] analysis of up-regulated and down-regulated mRNAs, as well as the analysis of genetic disease annotation. KEGG pathway analysis showed that 576 up-regulated mRNAs were enriched in 66 pathways, including fourteen pathways that might be related to CHD, such as “RNA polymerase”, “mTOR signaling pathway”, “Ras signaling pathway”, “focal adhesion”, “metabolic pathways”, etc. And 336 down-regulated mRNAs were enriched in 26 pathways, including nine pathways that might be related to CHD, such as “metabolic pathways”, “NOD-like receptor signaling pathway”, “cell adhesion molecules”, “MAPK signaling pathway”, etc. (Fig. 2B). According to the disease annotation in KOBAS database, 22 CHD-related DE mRNAs were identified, in which the proteins encoded by DNA damage inducible transcript 3 (DDIT3), sal-like protein 4 (SALL4), nuclear receptor interacting protein 1 (NRIP1), ankyrin repeat domain 6 and estrogen receptor alpha gene (ESR1) were transcription factors (TFs) (Additional file 1: Table S1).

### qRT-PCR validation of DE mRNAs associated with CHD

Twenty-two CHD-related DE mRNAs were validated by quantitative real-time PCR (qRT-PCR). The results showed that the expression of 18 genes was consistent with the results of chip detection, and the expression of two genes was opposite to the results of chip detection (NRIP1, ankrd6). There was no significant change in the expression of the two genes (flnb, GJB2) (Additional file 1: Fig. S1A, S1B).

### Protein and protein interaction analysis

**Fig. 3:**
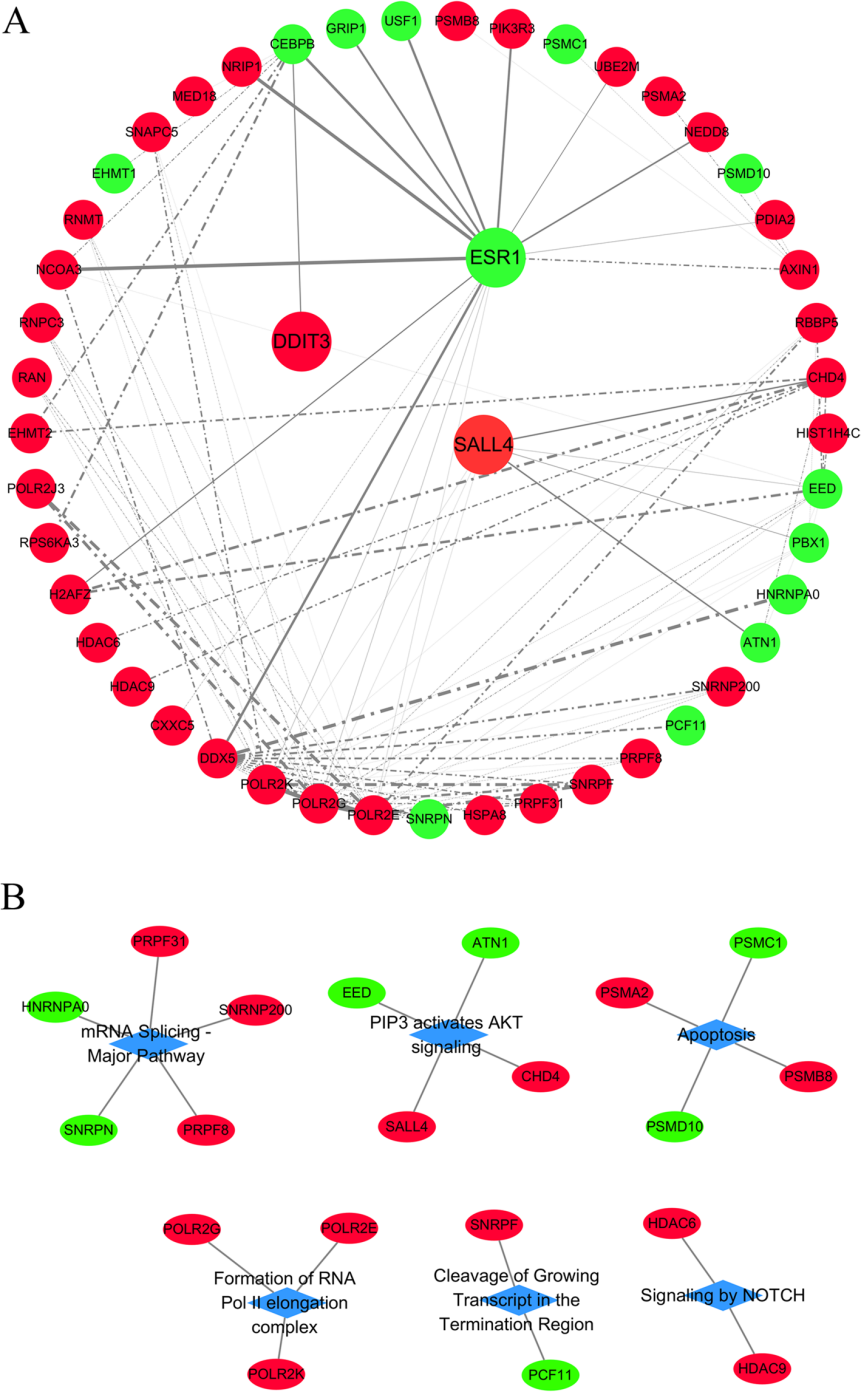
Construction of protein-protein interaction network. **A** PPI network of SALL4, DDIT3, ESR1 and their interacting proteins. The red dots and green dots represent represent up-regulated and down-regulated DE mRNAs and TFs, respectively. Solid lines represent positive relationship and dash lines represent negative relationship; Line thickness represents combine score. **B** Network of Reactome pathways and enriched proteins. Blue diamond represents pathway; Red and green elipse represents up-regulated and down-regulated mRNAs, respectively. PPI, protein-protein interaction; DDIT3, DNA damage inducible transcript 3; SALL4, sal-like protein 4; ESR1, estrogen receptor alpha gene; TF, transcription factor

There DE mRNA (DDIT3, SALL4 and ESR1) encoding TFs were selected for protein and protein interaction (PPI) analysis with all DE mRNAs using STRING database (minimum required interaction score ≥ 0.9). The results showed that there were seven DE mRNAs directly interacting with SALL4, one DE mRNA directly interacting with DDIT3, 15 DE mRNAs directly interacting with ESR1 and 27 DE mRNA indirectly interacted with these three TFs. These three TFs and their directly and indirectly interacting proteins were used to construct a network by Cytoscape (Fig. 3A). The Reactome (https://reactome.org/) database was used for interacting proteins for pathway analysis. The interacting proteins were mainly enriched in “mRNA splicing - major pathway” (FDR = 0.0000529), “PIP3 activates AKT signaling” (FDR = 0.0024), “formation of RNA Pol II elongation complex” (FDR = 0.0033), “apoptosis” (FDR = 0.0037), “signaling by NOTCH” (FDR = 0.028), “cleavage of growing transcript in the termination Region” (FDR = 0.041) (Fig. 3B).

### Coding and noncoding co-expression analysis of CHD related DE mRNAs

**Fig. 4:**
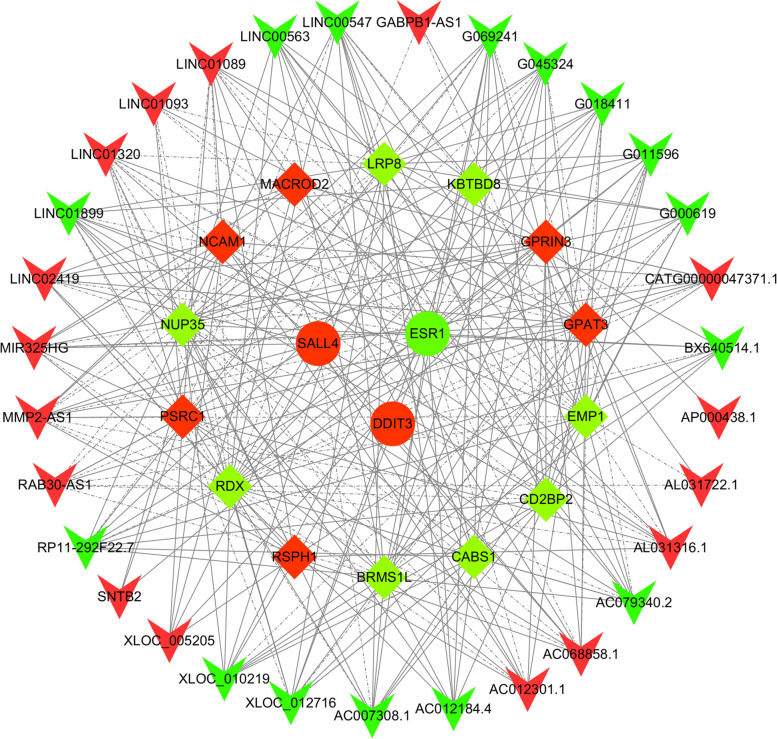
Construction of the CNC network. Red and green V represents up-regulated and down-regulated DE lncRNAs, respectively. Red diamond and ellipse represent up-regulated DE mRNAs associate with CHD; Green diamond and ellipse represent down-regulated DE mRNAs associate with CHD; Ellipse represent transcription factor; Solid lines represent positive relationship and dash lines represent negative relationship; Line thickness represents combine score. DE, differentially expressed; CNC, coding and non-coding co-expression; CHD, coronary heart disease

Top 20 up-regulated/down-regulated DE lncRNAs and 912 DE mRNAs were selected for coding and non-coding co-expression (CNC) analysis (Additional file 1: Table S2). Results of qRT-PCR showed that the expression of 18 CHD related mRNAs was consistent with the results of microarray results. Then, the 18 CHD related mRNAs were selected as the target genes to match the co-expressed lncRNA-mRNA pairs. A total of 299 lncRNA-mRNA co-expression pairs, which including 31 DE lncRNAs and 17 DE mRNAs, were identified. Among them, 221 lncRNA-mRNA pairs were positively co-expressed and 78 lncRNA-mRNA pairs were negatively co-expressed. We used Cytoscape v3.8.2 to construct CNC networks for these lncRNA-mRNA pairs (Fig. 4).

### Competing endogenous RNA analysis of CHD related DE mRNAs

**Fig. 5:**
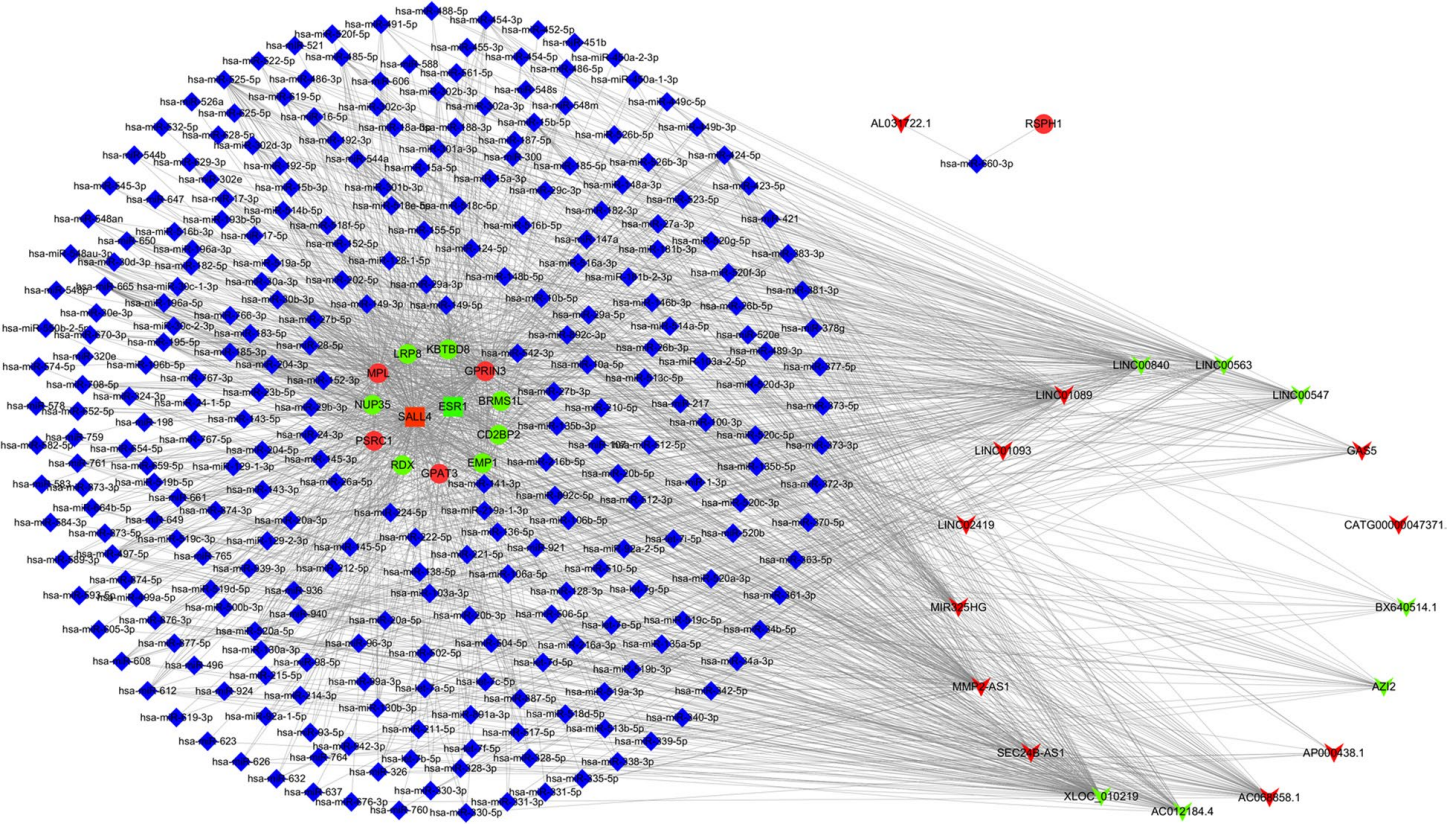
Construction of the ceRNA network. Red and green V represents up-regulated and down-regulated DE lncRNAs, respectively. Blue diamond represents microRNA; Red and green ellipse represent up-regulated and down-regulated DE mRNAs associate with CHD; Red and green rectangle represent up-regulated and down-regulated transcription factor associate with CHD. DE, differentially expressed; CHD, coronary heart disease

Based on TargetScan and miRanda database, top 20 up-regulated/down-regulated DE lncRNAs and 912 DE mRNAs were selected for competing endogenous RNA (ceRNA) analysis. Taking the CHD related DE mRNA verified to be consistent with the microarry results as the target gene, reverse search the lncRNAs that competitively binds with miRNA. As a result, there were 18 DE lncRNAs, 281 miRNAs and 14 DE mRNAs forming 1566 edges. Using Cytoscape v3.8.2 to drawn the ceRNA network (Fig. 5).

### qRT-PCR validation of DE lncRNAs

Thirty-one DE lncRNAs that may be related to CHD were screened by CNC analysis. Eighteen DE lncRNAs that may be related to CHD were screened by ceRNA analysis. Venn diagram showed that there are 14 intersecting DE lncRNAs, including 9 up-regulated lncRNAs (AP000438.1, MMP2-AS1, AC068858.1, MIR325HG, LINC01089, LINC02419, CATG00000047371.1, LINC01093, AL031722.1) and 5 down-regulated lncRNAs (LINC00547, XLOC_010219, AC012184.4, LINC00563, BX640514.1) (Additional file 1: Fig. S2A).

These 14 DE lncRNAs were validated by qRT-PCR. The results showed that the expression of 8 DE lncRNAs were consistent with the microarray results. The expression of AP000438.1, MMP2-AS1, AC068858.1, LINC02419 and LINC01093 were significantly up-regulated. The expression of LINC00547, AC012184.4 and BX640514.1 were significantly down-regulated. But the expression of other DE lncRNAs did not change significantly (Additional file 1: Fig. S2B). Next, we do bioinformatic analyses of these eight DE lncRNAs.

### Coding and noncoding co-expression and competing endogenous RNA analysis of target DE lncRNAs

**Fig. 6:**
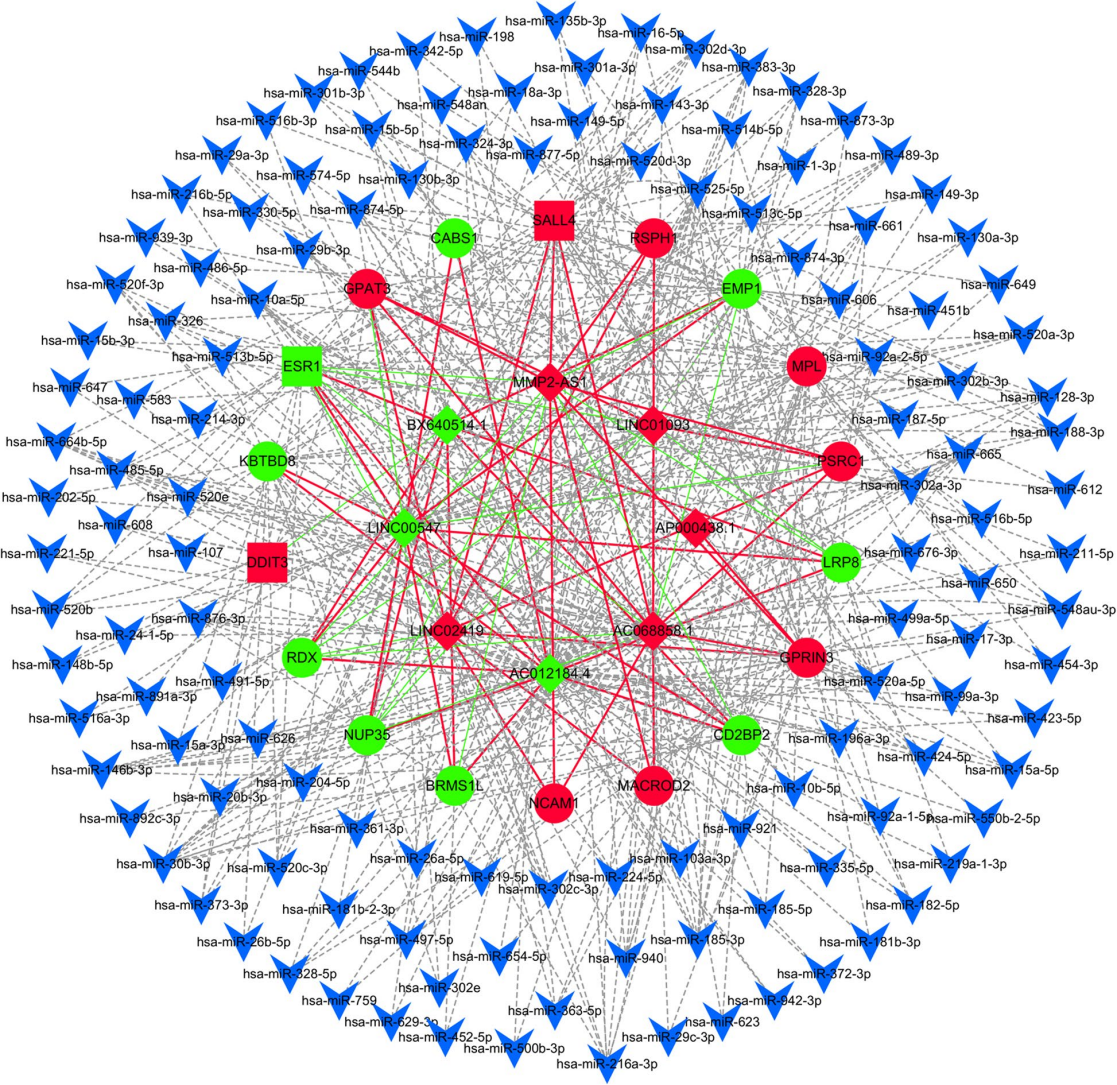
Construction of the ceRNA network of 8 DE lncRNAs. Red and green diamond represents up-regulated and down-regulated DE lncRNAs, respectively. Blue V represents microRNA. Red and green ellipse represent up-regulated and down-regulated DE mRNAs associate with CHD; Red and green rectangle represent up-regulated and down-regulated transcription factor associate with CHD. Red solid lines represent positive relationship and lncRNA-mRNA coexpression. Green solid represent negative relationship and lncRNA-mRNA coexpression. Line thickness represents combine score. Dash dot represent lncRNA-microRNA-mRNA competitive combination

Eight DE lncRNAs and 18 CHD-related DE mRNAs consistent with microarray results were selected to perform CNC and ceRNA analysis again. The Pearson correlation coefficients values |r| ≥ 0.9 and *P* < 0.001 were filtered out to construct CNC network. Seventy lncRNA-mRNA coexpression pairs were obtained from CNC analysis, including 48 positively and 22 negatively regulated coexpression pairs. And ceRNA miRNA coverage≥0.1, context+ < − 0.1, structure> 140, *P* < 0.01. A total of 146 nodes and 207 links between 7 DE lncRNAs, 11 DE mRNAs and 128 microRNAs were obtained from ceRNA analysis (Fig. 6).

### Subcellular localization analysis of target DE lncRNAs

We used lncRNA target genes to infer the subcellular location. Subcellular localization analysis showed that lncRNA AP000438.1, MMP2-AS1, AC068858.1, LINC00547, AC012184.4, LINC01093 and BX640514.1 are located in cytoplasm or cytoplasmic matrix. The localization information given by lncLocater and iLoc-LncRNA databases is the same. However, analysis using lncLocater database showed that LINC02419 was located in the ribosome, and analysis using iLoc-LncRNA database showed that LINC02419 was located in the cytoplasm (Additional file 1: Fig. S3).

### Prediction of m^6^A site about target DE LncRNAs

N6-methyladenosine (m^6^A) modification sites of 8 DE lncRNA were predicted based on Drach motif through sRAMP database. The locus with the highest comprehensive score was selected for display (Additional file 1: Fig. S4).

**Fig. 7:**
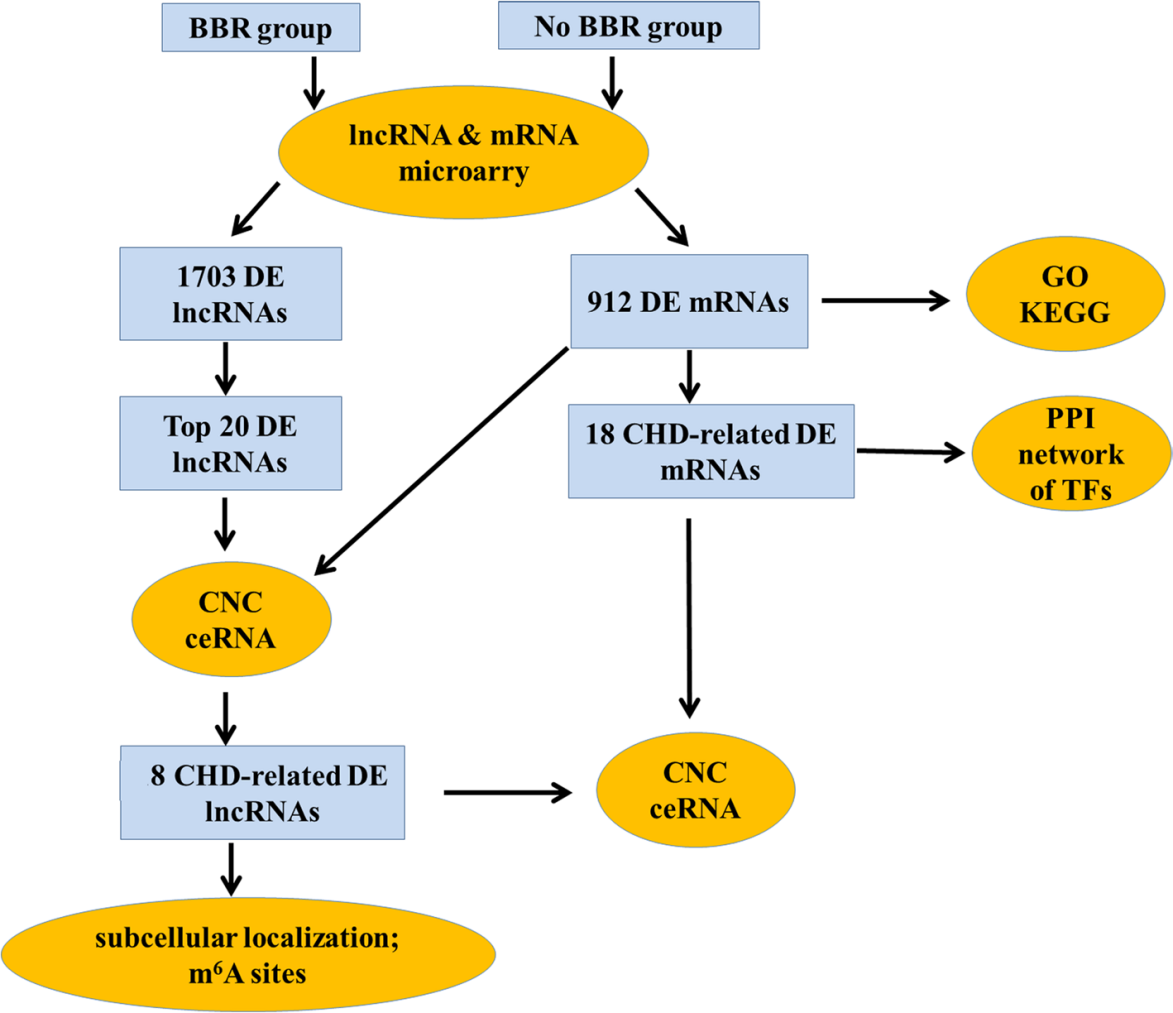
Flow chart of the approach utilized in the present study

In this study, we first detect the lncRNAs and mRNAs expression profile in whole blood samples. We performed GO analysis to determine the functional significance of 912 DE mRNAs. According to the disease annotation in KOBAS database, 22 CHD-related DE mRNAs were identified in which 3 proteins were TFs. PPI analysis were constructed between these TFs with 912 DE mRNAs. We selected the top 20 up-regulated/down-regulated DE lncRNAs for CNC analysis and ceRNA analysis with 921 DE mRNAs. Then, we identified 8 DE lncRNAs that may relate to CHD verified by the qRT-PCR. At last, we performed CNC and ceRNA mechanism analysis of these 8 DE lncRNAs and CHD-related DE mRNA, and predicted their subcellular localization and m^6^A modification sites**.** The flowchart of data collection and method implementation show in Fig. 7.

## Discussion

In order to understand pharmacological mechanism of BBR in cardiovascular diseases, we analyzed the changes of lncRNA and mRNA expression profiles in patients with stable CHD by microarray analysis. This result indicated that there are significant differences in the expression patterns of lncRNAs and mRNAs after taking BBR.

### Biological function analyses of DE mRNAs

We first performed GO and KEGG analyses of DE mRNAs. KEGG analyses could be identifying relevant functional pathways. KEGG analysis was performed on 912 DE mRNAs, and we found that “mTOR signaling pathway” and “MAPK signaling pathway” were significantly enriched. Abnormalities of “mTOR signaling pathway” and “MAPK signaling pathway” are associated with a variety of diseases, such as type 2 diabetes, cancer, neurodegenerative diseases, cardiovascular diseases, and autoimmune diseases [15]. Elevated serum miR-3129-5p in patients with CHD might contribute to CHD by targeting mTOR signaling [16]. Leucine induces cardioprotection in cardiac myocytes isolated from adult male Wistar rats by promoting mitochondrial function via mTOR signaling pathway [17]. In a mouse model of MI induced by surgical ligation of the left anterior descending coronary artery, IKKε protected the survival of myocardial cells by modulating the Akt/mTOR signaling pathway, and reduced MI-post autophagy [18]. Hu Y et al. found that cannabinoid receptor2 decreased the level of autophagy via AMPK-mTOR-p70S6K signaling in mice, leading to a detrimental effect of MI [19]. Zhou et al. found that miR-363-3p attenuate apoptosis, oxidative stress injury, and the inflammatory reaction via inactivation of the NOX4-dependent p38 MAPK axis in in isolated coronary arterial endothelial cells [20]. Dioscin may reduce oxidative stress and inflammation in CHD model pigs through Sirt1/Nrf2 and p38 MAPK pathways [21]. Moreover, many studies have found that BBR can regulate “mTOR signaling pathway” and “MAPK signaling pathway”. BBR inhibits activation of the APP/PS1 mice hippocampal mTOR/p70S6K signaling pathway, promotes amyloid-β degradation, and improves memory and spatial learning in APP/PS1 mice [22]. BBR improves chemo-sensitivity to cisplatin by promoting gastric cancer cell apoptosis and inhibiting the PI3K/AKT/mTOR signaling pathway [23]. BBR regulates the function of lysophosphatidic acid by blocking the lysophosphatidic acid-mediated P38/ERK MAPK pathway and inhibits proliferation and inflammation in patients with osteoarthritis [24]. BBR inhibits influenza A virus replication by blocking the MAPK/ERK pathway in lung epithelial type I cells and human alveolar basal epithelial cells from adenocarcinoma [25]. Our study suggested that BBR may modulate the expression of “mTOR signaling pathway” and “MAPK signaling pathway” in patients with CHD after took BBR orally for 3 months, and changes in these pathways may be involved in the healing process after CHD. Fortunately, some therapeutic drugs and pharmacologic compounds involving mTOR inhibitor have been reported to regulate apoptosis and autophagy [19].

### Biological function analyses of CHD-related TFs

According to disease annotations from the KOBAS database, validation of 22 CHD-related DE mRNAs by qRT-PCR revealed significant changes in TFs encoded by DDIT3, SALL4 and ESR1. We further performed PPI analysis of these TFs between DE mRNAs. PPI analysis can help us understand the direct relationship between these proteins. AKT plays an important role in cell survival signaling and in the regulation of apoptosis. Activated AKT inhibits the expression of the proapoptotic Bcl-2 family members Bad and Bax. SALL4 is a tumor promoting gene and is associated with the progression of many cancers, such as hepatocellular carcinoma, esophageal squamous cell carcinoma, acute myeloid leukemia and endometrial cancer. SALL4 exerts its tumor promoting effects through apoptotic pathways. SALL4 is highly expressed in breast cancer tissues. Knockdown SALL4 can induce cell apoptosis, suppress the cell proliferation, and reduce the invasion and migration ability of breast cancer cells [26]. Cheney et al. found that SALL4 was down-regulated in cell lines and glioma tissues. By down-regulating the expression of SALL4, miR-103, miR-195, and miR-15b suppressed the growth, migration, and invasion of glioma cells and increased cell apoptosis [26]. ESR1 also regulates apoptosis in a variety of tissues. CirC-0040039 may promote apoptosis of nucleus pulposus cells and aggravate intervertebral disk degeneration by inhibiting ESR1 expression and promoting miR-874-3p [27]. In primary rat pachytene spermatocytes, E2 can induce p38-dependent mitochondrion apoptotic pathway, c-Jun and ERK1/2 by activating GPER and ESR1 [28]. Studies have found that apoptosis caused by ischemic injury is a pathophysiological event in the process of CHD. Apoptosis plays a key role in myocardial cell loss in heart failure [29]. Reducing cardiomyocyte apoptosis is an important therapeutic target for CHD, but there is no effective method to avoid the apoptosis. Our findings suggest that BBR may affect downstream apoptosis pathways through DDIT3, SALL4 and ESR1, and AKT may be the signaling pathway for BBR to regulate the apoptosis. This study provides a new direction for reducing myocardial cell apoptosis in CHD.

### Biological function analyses of CHD-related mRNAs

Our study also selected the CHD-related DE mRNAs that were consistent with the microarray results as the target genes, and reversely looked for the DE lncRNAs that regulates them. These lncRNAs may be downstream target genes regulated by BBR. We found that GAS5 regulate CHD-related mRNAs through ceRNA mechanism. GAS5 is a lncRNA that is involved in the development of cardiovascular disease. Studies have found that GAS5 can attenuate cardiac fibrosis by regulating PTEN/MMP-2 signaling pathway and improve cardiac function by inhibiting Wnt/β-catenin signaling pathway [30, 31]. GAS5 expression was significantly down-regulated in the heart tissue of diabetic cardiomyopathy mice. Overexpression of GAS5 can inhibit NLRP3 inflammasome activation-mediated pyroptosis, improve cardiac function and myocardial hypertrophy in diabetic cardiomyopathy mice [32]. High expression of GAS5 in cardiomyocytes during hypoxia/reoxygenation can promote apoptosis induced by ischemia-reperfusion through miR-335/ROCK1/AKT/GSK-3β [33]. GAS5 is also one of the downstream target genes regulated by BBR. In the diabetic nephropathy model, positive feedback on the loop of C/EBPb/GAS5/miR-18a-5p was activated to reduce mitochondrial reactive oxygen species in HK-2 cells [34]. However, it has not been reported whether BBR participates in the repair process of CHD by regulating GAS5. In our microarray analysis, it was found for the first time that GAS5 may be the downstream target gene regulated by BBR on CHD and regulate CHD-related DE mRNAs. This provides new insights into the mechanism of BBR.

### Construction of the CNC network and ceRNA network to identify biological function analyses of DE lncRNAs

CNC analysis screened out 31 DE lncRNAs that may be related to CHD, and ceRNA analysis screened out 18 DE lncRNAs that may be related to CHD. A total of 14 DE lncRNAs were found in the intersection, and 8 of them were consistent with the microarry results after qRT-PCR verification. These 8 DE lncRNAs may be downstream target genes regulated by BBR and deserve further study in the future. It is still unclear whether BBR can participate in the progress of stable CHD by regulating these lncRNAs. We have observed a possible association between them and CHD by bioinformatics analysis. We performed CNC and ceRNA mechanism analysis of these 8 DE lncRNAs and CHD-related DE mRNA, and predicted their subcellular localization and m^6^A modification sites. These analyses can provide basic data for in-depth analysis in the future.

## Conclusion

Our research found that after taking BBR in patients with stable CHD, a large number of changes in the expression of lncRNAs and mRNAs molecules occurred. Some of the changes in molecular expression are related to the progression of stable CHD. Based on these findings, we suggested that BBR might become a novel therapeutic drug for stable CHD.

## Materials and methods

### Patients and sample collection

In the present study, peripheral whole blood samples were collected from 5 patients with stable CHD who took BBR orally for 3 months (300 mg BBR, Tid, Chengdu Jinhua Co.), and 5 patients with stable CHD who did not took BBR (aged 18–75 years) at Peking Union Medical College Hospital between March and December of 2019. The baseline demographic summary of 10 participants for microarray analysis was shown in Additional file 1: Table S3. Patients were excluded if they had uncontrolled hypertension, uncontrolled arrhythmia, adjusted the dose of vasodilators and statins within 4 weeks; plan to perform percutaneous coronary intervention or other invasive operators during the study period, treatment with BBR within 4 weeks; allergic to BBR, Cr > 1.5 mg/dL, ALT or bilirubin levels exceeding 3 times the upper limit of normal, heart failure or LVEF < 50%, pregnancy and lactation, malignant tumors or life expectancy less than half a year. This study was conducted according to the guidelines of the Declaration of Helsinki, and approved by the Peking Union Medical College Hospital Ethics Committee, and each patient provided signed informed consent for research purposes (No. JS-1500).

### LncRNA & mRNA microarray detection and analysis

Briefly, mRNA was purified from total RNA after the removal of rRNA (mRNA-ONLY™ Eukaryotic mRNA Isolation Kit, Epicentre, USA). RNA quantity and quality were measured on a NanoDrop ND-1000 (NanoDrop, USA). RNA integrity was assessed using standard denaturing agarose gel electrophoresis or an Agilent 2100 Bioanalyzer. Human lncRNA plus mRNA microarray v5.0 (Arraystar, 8 × 60 K, USA) was used for detection of differential expression of lncRNAs and mRNAs. Whole blood RNA was extracted and purified using TRIzol Reagent (Invitrogen, USA) and RNasey Mini Kit (Qiagen, German) from BBR group (*n* = 5) and control group (*n* = 5). Sample labeling and microarray hybridization were performed according to the Agilent One-Color Microarray-Based Gene Expression Analysis protocol (Agilent Technologies, USA) with minor modifications. Data were extracted with Feature Extraction version 11.0.1.1 (Agilent Technologies, USA). Quantile normalization and subsequent data processing were performed using the GeneSpring GX v12.1 software package (Agilent Technologies, USA). The threshold set for DE lncRNAs and mRNAs was *P* < 0.05, Fold-change ≥2.0. Violcano plot was conducted to explore signal intensity of DE lncRNAs and DE mRNAs. Hierarchical clustering was performed to display the distinguishable DE lncRNAs and DE mRNAs expression pattern among samples. All the microarray hybridization and analysis were carried out by Aksomics (Shanghai, China).

### Functional enrichment analysis of DE mRNAs

GO enrichment analysis was evaluated by Database for Annotation, Visualization and Integrated Discovery (https://david.ncifcrf.gov) to display the potential biological functions of DE mRNAs [35]. Pathway analysis was evaluated by KOBAS v3.0 bioinformatics tool (http://kobas.cbi.pku.edu.cn/) to identify significantly enriched signaling transduction pathways [36]. The significance of GO terms including BP, CC, MF and pathways were calculated by Fisher Exact test *P*-value and also −log10 (*P*-value) transformed as the enrichment score. The recommend *P*-value was cut off less than 0.05.

### Quantitative real-time PCR

To validate our microarray data and estimate the potential roles of the targeted DE mRNAs and DE lncRNAs, qRT-PCR experiments were performed using uperScriptTM III Reverse Transcriptase (Invitrogen, USA) and 2 × SYBR Green PCR Master Mix (Arraystar, USA) according to the manufacturer’s instructions. All data were normalized to GAPDH data to calculate the relative concentrations of lncRNAs and mRNAs. Details of the genes and primers were listed in Additional file 1: Table S4 and S5.

### Protein and protein interaction analysis

PPI analysis was performed based on STRING database (https://string-db.org/) [37]. According to the disease annotation in KOBAS database, 22 CHD-related DE mRNAs were identified, in which there proteins were TFs. Three DE mRNAs that encoded TF and their interacting proteins from all DE mRNAs we detected were used to construct PPI network by Cytoscape (v3.8.2) with minimum required interaction score ≥ 0.9. Then, the Reactome (https://reactome.org/) database was used for pathway analysis. The recommend *P*-value was cut off less than 0.05.

### LncRNA-mRNA co-expressed analysis

CNC analysis is to identify interactions between lncRNAs and mRNAs. We selected the top 20 up-regulated/down-regulated DE lncRNAs for CNC analysis with 921 DE mRNAs we detected. According to previous results, CHD related mRNAs verified to be consistent with the microarray results were selected as the target genes to match the co-expressed lncRNA-mRNA sub-pairs. The targeted DE mRNAs and DE lncRNA with Pearson correlation coefficients values |r| ≥ 0.9 and *P* < 0.001 were filtered out to construct CNC network by Cytoscape (v3.8.2).

### Construction of LncRNA-miRNA-mRNA ceRNA regulatory network

LncRNAs are known to competitively bind microRNAs (miRNAs) as competing endogenous RNAs (ceRNA) mechanism to affect the expression of targeted mRNAs. We used miRanda database (http://cbio.mskcc.org/miRNA2003/miranda.html) [38] and TargetScan database (http://www.targetscan.org/vert_72/) [39] to predict the potential miRNA-binding sites of top 20 up-regulated and down-regulated DE lncRNAs and all DE mRNAs. Then, related DE lncRNAs were filtered out through the DE mRNAs validated by qRT-PCR and overlapping miRNA binding. Targeted lncRNA-miRNA-mRNA ceRNA network were further constructed by Cytoscape (v3.8.2).

### Bioinformatic analysis of Targete DE LncRNAs

The function of lncRNA is closely related to their subcellular localization. In the nucleus, lncRNAs regulate gene expression at the epigenetic and transcriptional levels, and in the cytoplasm, they regulate gene expression at the posttranscriptional and translational levels. Subcellular localization analysis of targeted DE lncRNAs was performed by lncLocater (http://www.csbio.sjtu.edu.cn/bioinf/lncLocator/) [40] and iLoc-LncRNA (http://lin-group.cn/server/iLoc-LncRNA/citation.php) [41] database. SRAMP (http://www.cuilab.cn/sramp) database was used to predict the m^6^A modification sites with score ≥ 0.6 [42].

### Statistical analysis

Statistical analyses were carried out with the GraphPad Prism v5.0 package (GraphPad Sofware, Inc., USA). Data were expressed as mean ± standard error of the mean, and Student’s t test (Mann–Whitney U) was used to determine the difference between two groups. *P* < 0.05 was considered significant differences.

## Supplementary Information

**Additional file 1.**

## Data Availability

The data that support the findings of this study have been deposited in the NCBI Gene Expression Omnibus (GEO) Database under Accession Number GSE186019. The following secure token has been created to allow review of record GSE186019 while it remains in private status: apelmmqevnohrad.

## Abbreviations

BBR: Berberine
CHD: Coronary heart disease
MIAT: Myocardial infarction associated transcript
lncRNAs: Long noncoding RNAs
MI: Myocardial infarction
mTOR: mammalian target of rapamycin
MAPK: Mitogen-activated protein kinase
GAS5: Growth arrest-specific transcript 5
DE: Differentially expressed
GO: Gene ontology
BP: Biological process
CC: Cellular component
MF: Molecular function
KEGG: Kyoto Encyclopedia of Genes and Genomes
DDIT3: DNA damage inducible transcript 3
SALL4: Sal-like protein 4
NRIP1: Nuclear receptor interacting protein 1
ESR1: Estrogen receptor alpha gene
TF: Transcription factors
qRT-PCR: quantitative real-time PCR
PPI: Protein and protein interaction
CNC: Coding and non-coding co-expression
ceRNA: Competing endogenous RNA
m6A: N6-methyladenosine
