# Supplementary material for: Effect of berberine on global modulation of lncRNAs and mRNAs expression profiles in patients with stable coronary heart disease
Source: BMC Genomics. 2022 May 26;23:400. doi: 10.1186/s12864-022-08641-2 (PMC9134690; doi:10.1186/s12864-022-08641-2)

**Supplemental Material**

**Table S1.** DE mRNAs associated with CHD detected by microarray

**Table S2:** The detailed information of top20 up-regulated and down-regulated DE lncRNAs

**Table S3.** Characteristics of 10 study participants

**Table S4.** Primers for mRNAs associated with MI validated by qRT-PCR

**Table S5.** Primers for lncRNAs of CNC and ceRNA analysis intersection validated by qRT-PCR

**Figure S1.** Validation of DE mRNAs by qRT-PCR. (A) Up-regulated DE mRNAs associated with CHD. (B) Down-regulated DE mRNAs associated with CHD. *P < 0.05, **P < 0.01 vs Ctrl group, n = 5. DE, differentially expressed; BBR, berberine; Ctrl, control; CHD, coronary heart disease.

**Figure S2.** Validation of DE lncRNAs by qRT-PCR. (A) Venn diagram of CNC and ceRNA analysis. (B) qRT-PCR results of DE lncRNAs. t-test, ***P* < 0.005, *****P* < 0.001 vs Ctrl group, n = 5. DE, differentially expressed; CNC, coding and non-coding co-expression; ceRNA, competing endogenous RNAs.

**Figure S3.** Subcellular localization analysis of 8 DE lncRNAs.

**Figure S4.** The m^6^A modification site of 8 DE lncRNAs.

**Table S1.** DE mRNAs associated with CHD detected by microarray

| **Gene Symbol** | **Transcript_ID** | ***P*-value** | **Fold Change** | **Regulation** | **Locus** | **TF** | **RNA length** | **Disease** |
| --- | --- | --- | --- | --- | --- | --- | --- | --- |
| GPAT3 | ENST00000611707 | 0.009855088 | 3.2112467 | up | chr4:84457067-84527025:+ |  | 2633 | Myocardial Infarction |
| DDIT3 | ENST00000551116 | 0.022269392 | 2.6196479 | up | chr12:57910372-57914300:- | Yes | 1067 | Myocardial Infarction |
| FLNB | ENST00000490882 | 0.013489163 | 2.4351002 | up | chr3:57994127-58156501:+ |  | 8079 | Myocardial Infarction |
| GPRIN3 | ENST00000609438 | 0.00017668 | 2.4179288 | up | chr4:90157537-90229161:- |  | 14244 | Myocardial Infarction |
| GJB2 | ENST00000645189 | 0.000498755 | 2.3689408 | up | chr13:20761609-20767149:- |  | 2362 | Myocardial Infarction |
| NCAM1 | ENST00000619839 | 3.86528E-05 | 2.2979726 | up | chr11:112831997-113146109:+ |  | 2991 | Myocardial Infarction |
| RSPH1 | ENST00000291536 | 0.000393844 | 2.2917392 | up | chr21:43892596-43916464:- |  | 1430 | Myocardial Infarction |
| MACROD2 | ENST00000217246 | 9.54323E-05 | 2.1676497 | up | chr20:13976015-16033842:+ |  | 4994 | Myocardial Infarction |
| PSRC1 | ENST00000369909 | 0.002536613 | 2.1346823 | up | chr1:109822179-109825790:- |  | 1742 | Myocardial Infarction (Early onset) |
| SALL4 | ENST00000217086 | 4.67644E-05 | 2.1226308 | up | chr20:50398870-50419059:- | Yes | 5208 | Myocardial Infarction |
| NRIP1 | ENST00000400202 | 0.014515284 | 2.1015728 | up | chr21:16333556-16437321:- | Yes | 7671 | Myocardial Infarction |
| MPL | ENST00000372470 | 0.001572453 | 2.0854643 | up | chr1:43803520-43818443:+ |  | 1908 | Myocardial Infarction |
| CABS1 | ENST00000273936 | 0.036447673 | 0.346364297 | down | chr4:71200683-71202833:+ |  | 1532 | Myocardial Infarction |
| RDX | ENST00000405097 | 0.015488208 | 0.358819544 | down | chr11:110066286-110167437:- |  | 2761 | Myocardial Infarction |
| LRP8 | ENST00000306052 | 0.030083281 | 0.390668157 | down | chr1:53708036-53793686:- |  | 7648 | Myocardial Infarction |
| EMP1 | ENST00000256951 | 0.013857543 | 0.397232039 | down | chr12:13349650-13372873:+ |  | 5921 | Myocardial Infarction |
| BRMS1L | ENST00000216807 | 0.025946173 | 0.401620813 | down | chr14:36295524-36341169:+ |  | 2657 | Myocardial Infarction |
| NUP35 | ENST00000295119 | 0.006557203 | 0.416535892 | down | chr2:183989083-184026408:+ |  | 1633 | Myocardial Infarction |
| KBTBD8 | ENST00000417314 | 0.044385455 | 0.43659886 | down | chr3:67048731-67061634:+ |  | 4680 | Myocardial Infarction |
| ANKRD6 | ENST00000522441 | 0.005415805 | 0.488081799 | down | chr6:90272120-90341055:+ | Yes | 3157 | Myocardial Infarction |
| ESR1 | ENST00000440973 | 0.007024703 | 0.488644417 | down | chr6:152011631-152424409:+ | Yes | 6466 | Acute myocardial Infarction |
| CD2BP2 | ENST00000305596 | 0.04905909 | 0.489784491 | down | chr16:30362087-30366682:- |  | 3421 | Myocardial Infarction |

DE, differentially expressed; CHD, coronary heart disease; TF, transcription factors.

**Table S2:** The detailed information of top20 up-regulated and down-regulated DE lncRNAs

| **GeneSymbol** | **GeneID** | **Transcript_ID** | **Fold Change** | **P-value** | **Regulation** | **Locus** | **RNA length** | **Relationship** |
| --- | --- | --- | --- | --- | --- | --- | --- | --- |
| SEC24B-AS1 | ENSG00000247950 | ENST00000499359 | 16.3024962 | 0.010217751 | up | chr4:110351119-110354973:- | 957 | natural antisense |
| CATG00000047371.1 | CATG00000047371 | FTMT20200000483 | 11.470795 | 0.000447911 | up | chr1:12212611-12213371:- | 761 | intergenic |
| AL031316.1 | ENSG00000227591 | ENST00000445272 | 9.9348775 | 0.00057783 | up | chr1:209834709-209848592:- | 381 | intergenic |
| AP000438.1 | ENSG00000257002 | ENST00000543624 | 9.5355795 | 0.012850843 | up | chr11:62677018-62685833:+ | 749 | natural antisense |
| MMP2-AS1 | ENSG00000260135 | NR_147198 | 8.4250883 | 0.000958559 | up | chr16:55460709-55496209:- | 800 | intronic antisense |
| AC012301.1 | ENSG00000267146 | ENST00000586391 | 8.3073007 | 0.000716073 | up | chr18:54539899-54541201:- | 581 | intronic antisense |
| AC068858.1 | ENSG00000254654 | ENST00000525114 | 8.122017 | 0.000690784 | up | chr11:44995453-44999579:+ | 1239 | natural antisense |
| AL139022.1 | ENSG00000259118 | ENST00000556127 | 8.0504205 | 0.040787551 | up | chr14:65548752-65558583:+ | 486 | natural antisense |
| LINC01320 | ENSG00000228262 | ENST00000604250 | 8.0483373 | 0.001274083 | up | chr2:34902930-34947600:+ | 857 | intergenic |
| MIR325HG | ENSG00000280870 | NR_110401 | 7.6333387 | 0.000327674 | up | chrX:75878199-76234957:- | 1089 | intergenic |
| SNTB2 | ENSG00000168807 | ENST00000528525 | 7.5769754 | 0.000940737 | up | chr16:69221545-69304084:+ | 448 | bidirectional |
| LINC01089 | ENSG00000212694 | ENST00000429892 | 7.4505427 | 0.000686551 | up | chr12:122233173-122241390:- | 1521 | exon sense-overlapping |
| GAS5 | ENSG00000234741 | ENST00000454068 | 7.4238284 | 0.015961901 | up | chr1:173833049-173836953:- | 688 | intergenic |
| GABPB1-AS1 | ENSG00000244879 | ENST00000558593 | 6.9535363 | 0.026078675 | up | chr15:50648668-50659636:+ | 507 | intergenic |
| LINC02419 | ENSG00000256906 | ENST00000535487 | 6.5804603 | 0.000398968 | up | chr12:130554870-130557230:+ | 1713 | intergenic |
| LINC01093 | ENSG00000249173 | ENST00000506479 | 6.5645888 | 0.008524755 | up | chr4:185815025-185819918:- | 634 | intergenic |
| AC067930.1 | ENSG00000254144 | ENST00000517300 | 6.5378206 | 0.02735527 | up | chr8:144624280-144624570:+ | 291 | intergenic |
| XLOC_005205 | XLOC_005205 | TCONS_00011739 | 6.4022721 | 0.000139962 | up | chr6:24796944-24798091:+ | 1031 | natural antisense |
| RAB30-AS1 | ENSG00000246067 | ENST00000527627 | 6.3947631 | 0.001792731 | up | chr11:82783108-82817761:+ | 504 | intergenic |
| AL031722.1 | ENSG00000260541 | ENST00000570247 | 6.3842405 | 0.025033605 | up | chr16:1891021-1893548:- | 220 | exon sense-overlapping |
| AZI2 | ENSG00000163512 | ENST00000295748 | 9.0266432 | 0.006290402 | down | chr3:28363844-28390281:- | 3127 | bidirectional |
| XLOC_012716 | XLOC_012716 | TCONS_00026366 | 6.9738519 | 0.018067016 | down | chr18:71615130-71625153:+ | 819 | intergenic |
| SPATA1 | ENSG00000122432 | ENST00000460286 | 6.7797131 | 0.003633683 | down | chr1:85009885-85031877:+ | 776 | bidirectional |
| LINC00681 | ENSG00000255494 | ENST00000530150 | 5.5887746 | 0.012327307 | down | chr8:12651752-12675800:+ | 504 | intergenic |
| AC007308.1 | ENSG00000272600 | ENST00000608856 | 5.5136184 | 0.006042615 | down | chr22:21243494-21245502:- | 373 | natural antisense |
| G018411 | G018411 | T079757 | 5.4926192 | 0.024940347 | down | chr12:54991520-54992507:- | 491 | intergenic |
| LINC00547 | ENSG00000275226 | ENST00000612438 | 5.4446282 | 0.015531632 | down | chr13:38109077-38125673:+ | 3314 | intergenic |
| BX640514.1 | ENSG00000264490 | ENST00000583223 | 5.3295782 | 0.005835591 | down | chr20:61172797-61174714:+ | 1918 | intergenic |
| LINC01252 | ENSG00000247157 | ENST00000545239 | 5.0706771 | 0.014521476 | down | chr12:11708634-11709986:+ | 502 | intergenic |
| LINC01899 | ENSG00000265352 | ENST00000584810 | 5.0612088 | 0.011413122 | down | chr18:69446792-69449517:- | 337 | intergenic |
| XLOC_010219 | XLOC_010219 | TCONS_00020953 | 4.8467502 | 0.043392735 | down | chr12:120718114-120720608:- | 1295 | intergenic |
| AC079340.2 | ENSG00000251377 | ENST00000504785 | 4.8139943 | 0.018700296 | down | chr4:153143620-153146551:- | 448 | intergenic |
| G045324 | G045324 | T196603 | 4.6829961 | 0.01306441 | down | chr2:110809426-110821983:- | 2105 | intergenic |
| G069241 | G069241 | T296241 | 4.6635724 | 0.012120031 | down | chr6:3861951-3875194:+ | 5463 | intergenic |
| LINC00840 | ENSG00000226808 | ENST00000649239 | 4.5281753 | 0.006220367 | down | chr10:44340709-44370650:+ | 2203 | intergenic |
| RP11-292F22.7 | ENSG00000259942 | ENST00000563046 | 4.4906357 | 0.013887936 | down | chr10:47656084-47657233:+ | 1150 | intergenic |
| AC012184.4 | ENSG00000285710 | ENST00000650629 | 4.3477291 | 0.010898087 | down | chr16:70382988-70406069:- | 1193 | natural antisense |
| LINC00563 | ENSG00000261097 | ENST00000566370 | 4.3448988 | 0.029541803 | down | chr13:46870580-46871979:- | 1400 | intergenic |
| G011596 | G011596 | T049507 | 4.3224808 | 0.041437909 | down | chr10:121241794-121243609:- | 983 | intergenic |
| G000619 | G000619 | T003301 | 4.2665198 | 0.034000347 | down | chr1:12611617-12614099:- | 1116 | intergenic |

DE, differentially expressed.

**Table S3.** Characteristics of 10 study participants

| **Characteristics** | **BBR *(n= 5)*** | **Control *(n= 5)*** | ***P*-value** |
| --- | --- | --- | --- |
| Gender, male, *n* (%) | 5(100) | 5(100) | 1 |
| Age | 57.6(9.3) | 52.2(12.2) | 0.4531 |
| Smoking, *n* (%) | 4(80) | 1(20) | 0.0578 |
| Body mass index, kg/sq. m | 25.0(2.1) | 25.0(1.2) | 0.9963 |
| Drinking, *n* (%) | 0 (0) | 2(40) | 0.1138 |
| Family history of CHD, *n* (%) | 5(100) | 1(20) | 0.0098 |
| Diabetes | 0 (0) | 1(20) | 0.2918 |
| Hyperlipidemia | 5(100) | 4(80) | 0.2918 |

**Table S4.** Primers for mRNAs associated with MI validated by qRT-PCR

| **Gene** | **Primer** | **Tm (℃)** | **Production length (bp)** |
| --- | --- | --- | --- |
| GAPDH | F:5’GGGAAACTGTGGCGTGAT3’  R:5’GAGTGGGTGTCGCTGTTGA3’ | 60 | 299 |
| DDIT3 | F:5' GCCCTCACTCTCCAGATTCC 3’  R:5’ CGAGTCGCCTCTACTTCCCT 3’ | 60 | 232 |
| MPL | F:5' GACCTGTTATCAACTCCGATACACA 3’  R:5’ CAGAGCGGTCACCAAGGAGA 3’ | 60 | 235 |
| GPAT3 | F:5' GCTAACAGGGTTAAGTCTGCTA 3’  R:5’ TCACCTTTGCTCTCTTTAGTCC 3’ | 60 | 85 |
| FLNB | F:5' TGCTGAGTACATTCCTTTCGC 3’  R:5’ TGGGGTCCACAACATCCTT 3’ | 60 | 113 |
| GPRIN3 | F:5' CCCTACTGCTCAATCCTAAATC 3’  R:5’ TGGCTGTCTTGGTCTGTCTGT 3’ | 60 | 126 |
| GJB2 | F:5' CCGACGCAGAGCAAACCG 3’  R:5’ CAACGAGGATCATAATGCGAAA 3’ | 60 | 143 |
| NCAM1 | F:5'CAAGAGGAAGACGATGAGAAGT3’  R:5’ CAGCCTCGTCGTTCTTATCC 3’ | 60 | 85 |
| RSPH1 | F:5' CGGAGACGGGCAGTAAGTAT 3’  R:5’ TGTGGTTCAGGTGAATGAGCT 3’ | 60 | 81 |
| MACROD2 | F:5' AAGGCGGTGAAGTGACAGAT 3’  R:5’ ATCGGGATGATCTTGGTCA 3’ | 60 | 50 |
| PSRC1 | F:5' GTAAGGTTTATTGTGGATGAGAC 3’  R:5’ GCATTTGGGTCACTTCGGT 3’ | 60 | 140 |
| SALL4 | F:5' ACCCTGTTGTGTGGAACCAG 3’  R:5’ TGGAGACAGTGGCGTTATTC 3’ | 60 | 150 |
| NRIP1 | F:5' GAACTGTTCTCAGGACTCATTATT 3’  R:5’ TCAGTGTTCGTCTGTCTCCAA 3’ | 60 | 184 |
| CABS1 | F:5' AATCCCTCCTGCTCCTGAA 3’  R:5’ AACACTTAGGTCAATTTCGGTT 3’ | 60 | 91 |
| RDX | F:5' CCGCTCTCCGGAAAGTGATAAC 3’  R:5’ TTGATTGGTTTCGGCATTTTCT 3’ | 60 | 92 |
| LRP8 | F:5' CTGTTATCGGGATCATCGTG 3’  R:5’ GGGTTGTCAAAATTCATGCTTT 3’ | 60 | 115 |
| BRMS1L | F:5' ACGATTAAGTCAGGTGGATGC 3’  R:5’ GGTTCCAAGTATTCTGGTGCT 3’ | 60 | 69 |
| NUP35 | F:5' CCTCTTGTTGGAGTTACATCTAC 3’  R:5’ CCCAAATACAGTCACCCAAG 3’ | 60 | 177 |
| KBTBD8 | F:5' ATGCTGCCCACAAACACTC 3’  R:5’ TAAACACCCTTCCTGTGACTAT 3’ | 60 | 72 |
| ANKRD6 | F:5' AAGGATGGGAAAGTGATGC 3’  R:5’ GATTAGAGGTTCACATCGACAA 3’ | 60 | 57 |
| CD2BP2 | F:5' AAGGGAACGGTTGGCTATG 3’  R:5’ CATCACATCCACCAGACCATC 3’ | 60 | 184 |
| ESR1 | F:5' TAGAGATCCTGATGATTGGTCT 3’  R:5’AGCAAGTTAGGAGCAAACAGTA 3’ | 60 | 76 |
| EMP1 | F:5' TCTTCTGTGTCATTGCCCTC 3’  R:5’ CTGCGTTCCATCACGATTC 3’ | 60 | 164 |

**Table S5.** Primers for lncRNAs of CNC and ceRNA analysis intersection validated by qRT-PCR

| **Gene** | **Primer** | **Tm(℃)** | **Production length (bp)** |
| --- | --- | --- | --- |
| GAPDH | F:5’GGGAAACTGTGGCGTGAT3’  R:5’GAGTGGGTGTCGCTGTTGA3’ | 60 | 299 |
| ENST00000543624 | F:5' GAAGAGGGTGGGTCTACTGGAG 3’  R:5’ TGAGACAGACGCAGCCCTTA 3’ | 60 | 59 |
| NR_147198 | F:5'ACTACTATGTTGTGAGCAGCCC3’  R:5’TCATCACTGCATTTCTCAAGGT3’ | 60 | 135 |
| ENST00000525114 | F:5' GCACCATTGAGACCACAGGA 3’  R:5’ AGCAGCAGGGGTGACAAGAG 3’ | 60 | 87 |
| NR_110401 | F:5'ACTGACATGAGCAGAGGAGGAT3’  R:5’ TCAACTGCTTCTTCATCCCAA 3’ | 60 | 156 |
| ENST00000429892 | F:5' TCTTCTAGACTCCATGGCACCG 3’  R:5’ GGCACTCTGCTAGAGGTTGAGG 3’ | 60 | 200 |
| ENST00000612438 | F:5' TGCTTCAGAAAGTGCGAGTC 3’  R:5’ GAGGTTCCCAAAACTCAGTTCT 3’ | 60 | 96 |
| TCONS_00020953 | F:5' TTCCCACAAATGATACACACG 3’  R:5’ TATGTAGCCACAGAAAAGATCC 3’ | 60 | 115 |
| ENST00000650629 | F:5' ACCGGAAGAATGAAGCACAC 3’  R:5’ CAGAGGAAACCAGGCAAAC 3’ | 60 | 70 |
| ENST00000566370 | F:5' TGTTATGTCACATGGCAATGG 3’  R:5’ ATTGGACCCACCCAGATGA 3’ | 60 | 110 |
| ENST00000535487 | F:5' GCAGGTAGCCATCCACAGTA 3’  R:5’ GCCCAAATGTCTTTATGGCCC 3’ | 60 | 101 |
| ENST00000583223 | F:5' GCAATTTGGAATGAGGGTGTTA3’  R:5’ GCTCTAGTCTGTGGAGGGCATA 3’ | 60 | 106 |
| FTMT20200000483 | F:5' CTTCTTGAAGTAGCTGGCAGTG3’  R:5’ GTCCAGTTTCTTGGGAGTGAG3’ | 60 | 91 |
| ENST00000506479 | F:5' CAGGTCGGGTGAAGAAGAGC 3’  R:5’ TCCATCACCCACGCACTATC 3’ | 60 | 130 |
| ENST00000570247 | F:5' CGATCGACCAGTCTCAACCTCT 3’  R:5’ CTCCTGGGTTCAAGCAATTCTC 3’ | 60 | 67 |

**Figure S1.** Validation of DE mRNAs by qRT-PCR. (A) Up-regulated DE mRNAs associated with CHD. (B) Down-regulated DE mRNAs associated with CHD. **P* < 0.05, ***P* < 0.01 vs Ctrl group, n = 5. + consistent with the results of chip detection, - opposite to the results of chip detection. DE, differentially expressed; BBR, berberine; Ctrl, control; CHD, coronary heart disease.


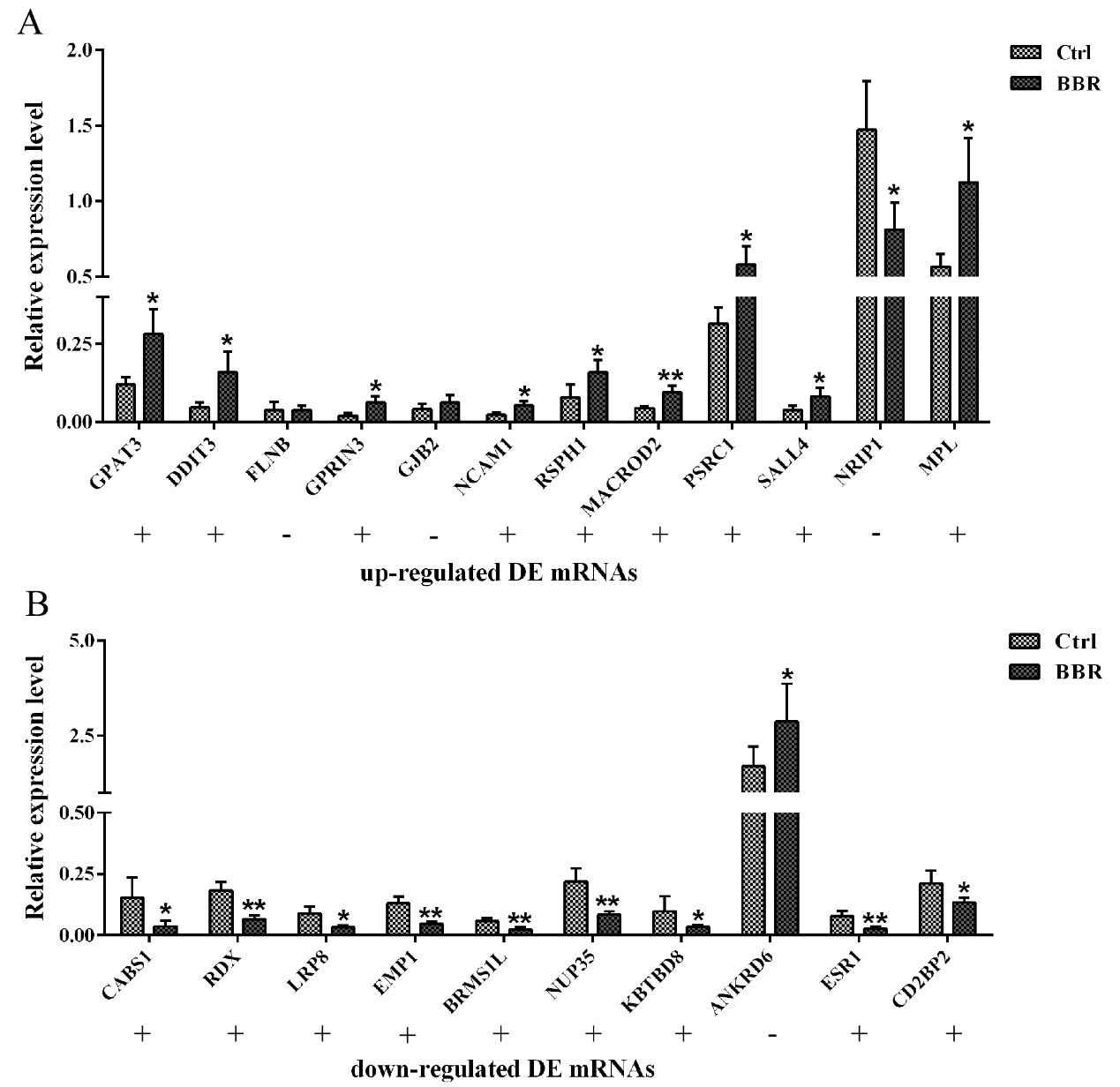


**Figure S2.** Validation of DE lncRNAs by qRT-PCR. (A) Venn diagram of CNC and ceRNA analysis. (B) qRT-PCR results of DE lncRNAs. t-test, ***P* < 0.005, *****P* < 0.001 vs Ctrl group, n = 5. DE, differentially expressed; CNC, coding and non-coding co-expression; ceRNA, competing endogenous RNAs.


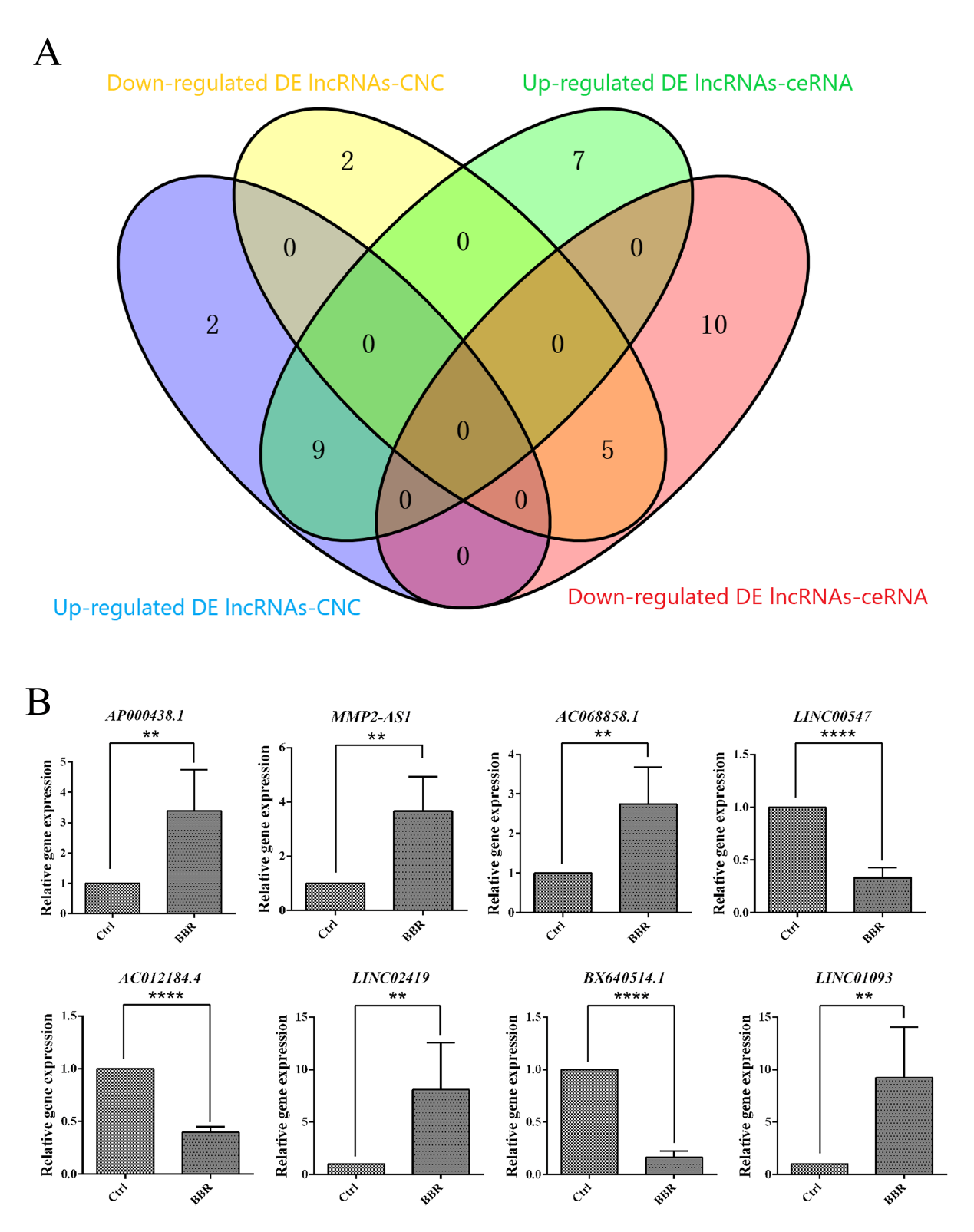


**Figure S3.** Subcellular localization analysis of 8 DE lncRNAs.

**
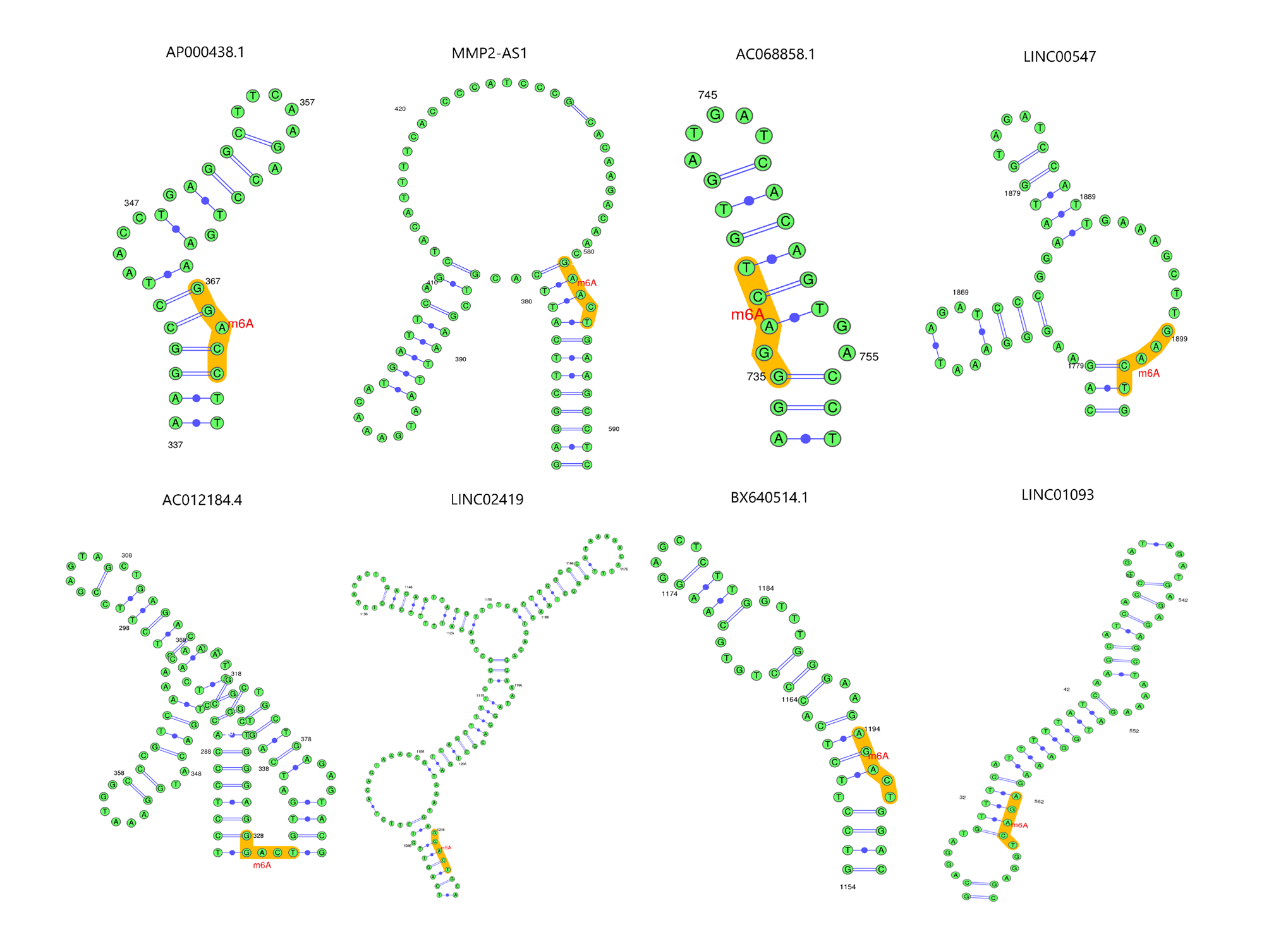
**

**Figure S4.** The m^6^A modification site of 8 DE lncRNAs.


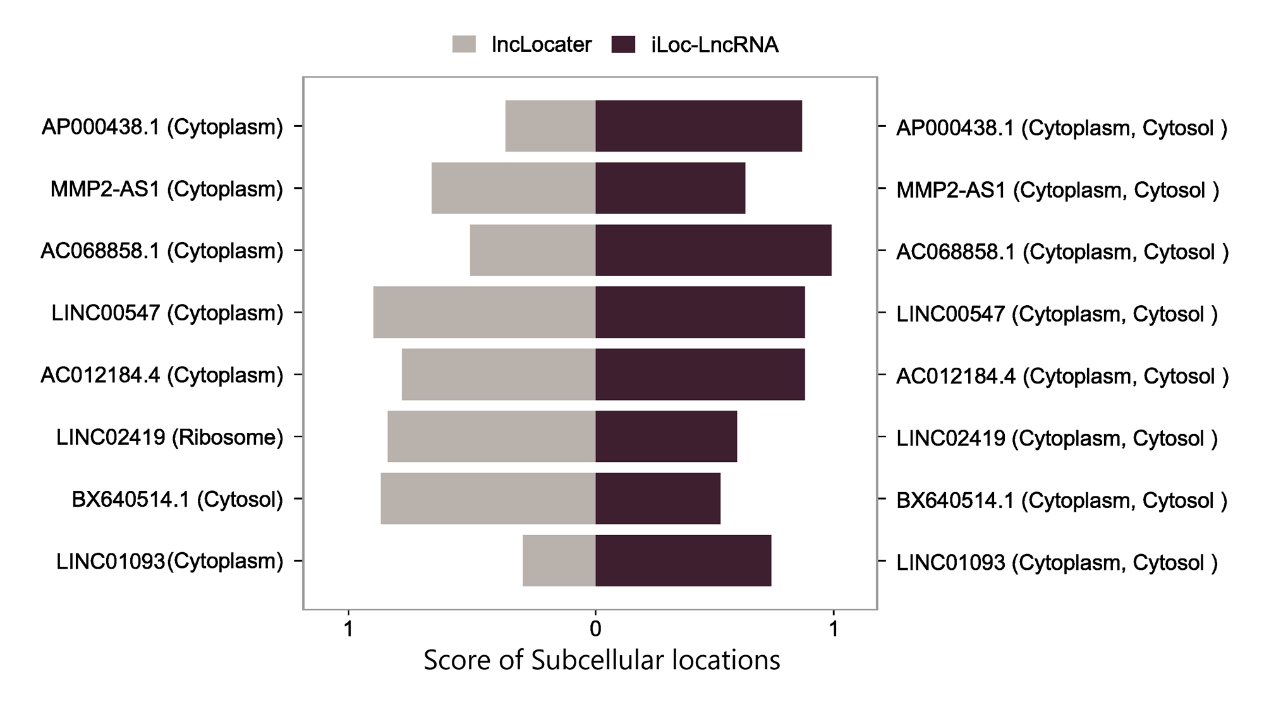

Supplement: Supplementary file 1 — Additional file 1. [file 12864_2022_8641_MOESM1_ESM.docx]
